# A Novel Form of Progressive Retinal Atrophy in Swedish Vallhund Dogs

**DOI:** 10.1371/journal.pone.0106610

**Published:** 2014-09-08

**Authors:** Ann E. Cooper, Saija Ahonen, Jessica S. Rowlan, Alison Duncan, Eija H. Seppälä, Päivi Vanhapelto, Hannes Lohi, András M. Komáromy

**Affiliations:** 1 Department of Small Animal Clinical Sciences, College of Veterinary Medicine, Michigan State University, East Lansing, Michigan, United States of America; 2 Department of Clinical Studies, School of Veterinary Medicine, University of Pennsylvania, Philadelphia, Pennsylvania, United States of America; 3 Gavin Herbert Eye Institute, Ophthalmology Research, University of California Irvine, Irvine, California, United States of America; 4 Department of Veterinary Biosciences and Research Programs Unit, Molecular Neurology, University of Helsinki, Helsinki, Finland and the Folkhälsan Institute of Genetics, Helsinki, Finland; 5 Department of Ophthalmology, University of Washington Medical School, Seattle, Washington, United States of America; 6 Veterinary Clinic Vetset, Kirkkonummi, Finland; Justus-Liebig-University Giessen, Germany

## Abstract

Inherited retinal degenerations, such as retinitis pigmentosa (RP) and age-related macular degeneration (AMD), represent leading causes of incurable blindness in humans. This is also true in dogs, where the term *progressive retinal atrophy (PRA)* is used to describe inherited photoreceptor degeneration resulting in progressive vision loss. Because of the similarities in ocular anatomy, including the presence of a cone photoreceptor-rich central retinal region, and the close genotype-phenotype correlation, canine models contribute significantly to the understanding of retinal disease mechanisms and the development of new therapies. The screening of the pure-bred dog population for new forms of PRA represents an important strategy to establish new large animal models. By examining 324 dogs of the Swedish vallhund breed in seven countries and across three continents, we were able to describe a new and unique form of PRA characterized by the multifocal appearance of red and brown discoloration of the tapetal fundus followed over time by thinning of the retina. We propose three stages of the disease based on the appearance of the ocular fundus and associated visual deficits. Electroretinography revealed a gradual loss of both rod and cone photoreceptor-mediated function in *Stages 2* and *3* of the disease. In the few dogs that suffered from pronounced vision loss, night-blindness occurred first in late *Stage 2*, followed by decreased day-vision in *Stage 3*. Histologic examinations confirmed the loss of photoreceptor cells at *Stage 3*, which was associated with the accumulation of autofluorescent material in the adjacent retinal pigment epithelium. Pedigree analysis was suggestive of an autosomal-recessive mode of inheritance. Mutations in six known canine retinal degeneration genes as well as hypovitaminosis E were excluded as causes of the disease. The observed variability in the age of disease onset and rate of progression suggest the presence of genetic and/or environmental disease modifiers.

## Introduction

Inherited retinal diseases are among the leading causes for incurable vision loss in the human and canine populations [1]–[5]. In dogs, most of these conditions are classified as *progressive retinal atrophy (PRA)*, and, similar to many forms of retinitis pigmentosa (RP) in human patients, primarily affect rod photoreceptors leading to initial night-vision loss followed by loss of cone photoreceptors and subsequent day-blindness [1], [3], [5]. Cone-rod dystrophies (crd) and primary cone degeneration (cd) have also been described in dogs with either combined initial day- and night-vision loss or exclusive day-blindness [1], [5]. Inherited retinal disease can also result from a primary dysfunction of the retinal pigment epithelium (RPE) of which *RPE65*- or *BEST1*-mutant dogs are examples [1], [5]–[9]. Because of the similarities in ocular anatomy, including the presence of a cone photoreceptor-rich central retinal region [10], [11], and the frequently similar genotype-phenotype correlation [1], canine retinal disease models contribute significantly to our understanding of retinal disease mechanisms and the development of new therapies for human patients [12]–[20]. Screening of the pure-bred dog population for new forms of PRA represents an important strategy to maintain breed health and to establish new large animal models.

**Figure 1:**
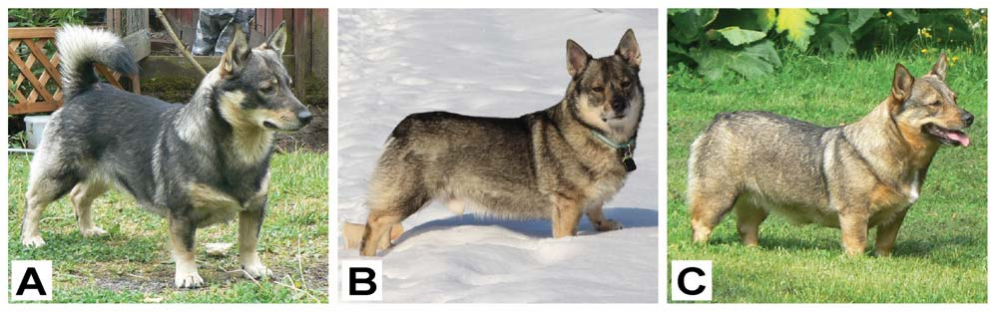
Appearance of Swedish vallhund dogs. The Swedish vallhund is a spitz-type herding breed, recognized since the age of the Vikings (700–1000 A.D.). The breed standard includes erect ears and a medium-length, hard haircoat. Coat color is a sable pattern in grey, red or combinations thereof. Tail may be natural (long, stub or bob) or docked.

Beginning in the late 1990s, Swedish and Finnish eye panelists recognized the emergence of a new retinal disease in Swedish vallhund dogs (**Fig. 1**). The morphologic retinal abnormalities seen by indirect ophthalmoscopy were different from any known forms of canine inherited retinopathy described to date (Finnish Kennel Club: http://jalostus.kennelliitto.fi/frmEtusivu.aspx; Swedish Kennel Club: http://kennet.skk.se/avelsdata/). With the gradual increase of the breed's international popularity, this Swedish vallhund retinopathy was also recognized in other parts of the world, including North America [2]. The purpose of this study was to characterize the phenotype and potential etiology of this condition in order to provide the basis for elimination of the disease in the canine population. Our findings are based on the examination of 324 Swedish vallhund dogs in seven countries and suggest that the Swedish vallhund retinopathy is a new form of PRA. An autosomal recessive mode of inheritance is most likely.

## Materials and Methods

### Ethics Statement

All of the described work was conducted in compliance with the ARVO Statement for the Use of Animals in Ophthalmic and Vision Research. Approval from the local Institutional Animal Care and Use Committee was obtained when the clinical examination or sample collection was not part of the services requested by the dog owner or breeder (County Administrative Board of Southern Finland ESLH-2009-07827/Ym-23 and University of Pennsylvania IACUC protocols# 802604 and 803429).

### Study Animals

Most of the results described in this study were obtained from Swedish vallhund dogs clinically examined by the authors (AMK and PV). The bulk of these dogs resided either in the United States or Finland, but additional dogs were also examined in Canada and Sweden. In addition, for pedigree and genetic analyses, Swedish vallhund dogs examined by other veterinary ophthalmologists or eye panelists were also included. These latter dogs resided in the United States, Canada, Finland, Sweden, the United Kingdom, the Netherlands, and Australia. Although other ocular and adnexal abnormalities such as persistent pupillary membranes and cataracts were noted, this study focused exclusively on retinal lesions.

### Clinical Examination

All dogs were examined by hand-held slit-lamp biomicroscopy (either SL-14, Kowa Company, Tokyo, Japan; or HSO 10, Carl Zeiss Meditec AG, Jena, Germany) and binocular indirect ophthalmoscopy (Keeler All Pupil II; Keeler Instruments, Broomall, PA, USA) with a condensing lens (Pan Retinal 2.2D; Volk Optical, Mentor, OH, USA) under pharmacologic mydriasis with topical 1% tropicamide ophthalmic solution. All ocular abnormalities were documented and, for selected dogs, fundus photographs were taken with either a portable camera (RC-2, Kowa Company, Tokyo, Japan) or through a 20-diopter lens (Volk Optical, Mentor, OH, USA) with a digital video camera mounted on an indirect ophthalmoscope (Omega 2C, Heine Optotechnik, Herrsching, Germany). Images were visualized with Pinnacle studio plus software (Pinnacle, Mountain View, CA, USA) and individual frames extracted using Vegas Movie Studio HD Platinum 11.0 (Sony Creative Software, Inc, Middleton, WI, USA). Additionally, visual performance was assessed by distribution of a short questionnaire among 40 owners of PRA-affected Swedish vallhund dogs in Finland. Specifically, we inquired as to the owner's perception of the onset and severity of visual deficits observed in the dogs, such as the ability to see stationary or moving objects under different light conditions.

### Electroretinography

Retinal function was recorded by electroretinography under general anesthesia in a total of 15 selected Swedish vallhund dogs in the United States, Sweden and Finland, using the same equipment and protocol (RETIport, Acrivet, Inc., Salt Lake City, UT, USA). Premedication by intramuscular injection occurred with acepromazine maleate (0.5 mg/kg) alone, or a combination of butorphanol tartrate (0.1–0.2 mg/kg) with either acepromazine maleate (0.01–0.02 mg/kg) or medetomidine HCl (20 µg/kg). While one dog was masked with an isoflurane/O_2_ gas mixture, the remaining dogs were induced by intravenous injection of propofol (4–6 mg/kg to effect). All dogs were intubated and inhalation anesthesia maintained with an isoflurane/O_2_ gas mixture on a semiclosed system. The dogs were placed in right lateral recumbency and left eyes were tested. Central position of the globe was achieved and maintained by retrobulbar injection of sterile saline caudal to the orbital ligament (volume to effect). In order to avoid muscle artifacts in the electroretinogram (ERG), an auriculopalpebral nerve block was applied by subcutaneous injection of 2% lidocaine HCl solution along the zygomatic arch. Two platinum subdermal needle electrodes (Grass Safelead Needle electrodes, Grass Technologies, West Warwick, RI, USA) were used: The reference electrode was placed subcutaneously approximately 10 mm from the lateral canthus, and the ground electrode was placed over the occipital protuberance [21]. A Kooijman/Damhof ERG corneal contact lens with built-in white LED light source (Acrivet, Inc.) was used as the active electrode and applied with 2.5% hypromellose ophthalmic demulcent solution [22]. Following 20 minutes of dark adaptation, a single rod and maximal rod-cone responses were recorded with a flash intensity of 0.03 cd.s/m^2^ (average of 20 sweeps at 0.2 Hz) or 3.0 cd.s/m^2^ (average of 10 sweeps at 0.06 Hz) respectively. Subsequently, the eyes were light-adapted to a white uniform background light of 25 cd/m^2^ from the Kooijman/Damhof ERG contact lens, and single cone (average of 10 sweeps at 0.5 Hz) and cone flicker (average of 30 sweeps at 28 Hz) responses were recorded with a 3.0 cd.s/m^2^ flash intensity. For all recordings, the filters were set to allow a bandpass of 1 to 300 Hz. For each recording, amplitudes and implicit times were measured and compared between individual dogs. The small sample size precluded detailed statistical analyses.

### Histopathology and Immunohistochemistry

Ocular histopathologic examination was performed in eight Swedish vallhund dogs that were euthanized for unrelated reasons, and the eyes donated by the owners. Most of the eyes were fixed in formalin, and routinely processed for paraffin embedding and hematoxylin and eosin (H&E) staining. For specific examination of lipopigment accumulation (ceroid/lipofuscin), tissue sections were stained with acid-fast and counterstained with hematoxylin. One eye was fixed for 24 h in 4% paraformaldehyde in 0.1 M phosphate-buffered saline (PBS) at 4°C. Then the anterior segment and the vitreous were removed, and the tissue was placed sequentially in 15 and 30% sucrose for 24 h each prior to embedding in optimal cutting temperature (OCT) medium for cryosectioning. Paraffin sections of normal and PRA-affected eyes originating from other canine breeds were provided as control samples by the Anatomic Pathology/Biopsy Service of the University of Pennsylvania School of Veterinary Medicine and the Comparative Ocular Pathology Laboratory of Wisconsin (COPLOW).

To further examine the morphology of cells and the localization of protein expression within the retina, immunohistochemical staining of both paraffin and OCT retinal sections was performed with the following antibodies (**Table S1**): human cone arrestin (for cone photoreceptors), rhodopsin (for rod photoreceptors), RPE65 (for the retinal pigment epithelium, RPE), glial fibrillary acidic protein (GFAP, for astrocytes and Müller cells), glutamine synthetase (for Müller cells) and G_0_ *alpha* (for ON bipolar cells). Alexa Fluor-labeled goat anti-rabbit immunoglobulin (IgG) or goat anti-mouse IgG (Molecular Probes Inc., Eugene, OR, USA) was used as secondary antibody. 4′,6-Diamidino-2-phenylindole (DAPI) stained the cell nuclei.

Stained tissues were evaluated using light and fluorescent microscopy with a Zeiss Axioplan microscope (Carl Zeiss Meditec, Dublin, CA, USA). Images were digitally captured (Spot 4.0 camera; Diagnostic Instruments Inc., Sterling Heights, MI, USA), and imported into a graphics program (Photoshop; Adobe, Mountain View, CA, USA) for display. Selected tissues were imaged by laser scanning confocal microscopy (Olympus FluoView FV1000; Olympus America, Center Valley, PA, USA).

### Pedigree and Candidate Gene Analysis

In an attempt to determine genealogical relationships between as many of the examined Swedish vallhund dogs as possible, a master pedigree was constructed using GenoPro genealogy software (http://www.genepro.com/). Genealogical data was either accessed in public canine registries such as the Finnish Kennel Club's Koiranet (http://jalostus.kennelliitto.fi/frmEtusivu.aspx) or from pedigree information provided by the dog owners.

When the owner's consent was obtained, either whole blood (in EDTA-containing tubes) or buccal swabs (Eurotubo Cytobrush, sterile, 200 mm, Danlab, Helsinki, Finland) were collected for genomic DNA extraction (QIAmp DNABlood Mini Kit, Qiagen, Valencia, CA, USA, or Chemagic Magnetic Separation Module I, Chemagen Biopolymer-Technologie AG, Baeswieler, Germany for whole blood; or QIAamp DNA Mini Kit, Qiagen for buccal swabs). DNA concentration was measured using Nanodrop ND-1000 UV/Vis Spectrophotometer (Nanodrop technologies, Wilmington, Delaware, USA); and the DNA was stored at -20°C. Genes associated with the following forms of inherited canine retinal diseases were tested for association using fragment analysis in 11 PRA-affected and 11 unaffected Swedish vallhund dogs: canine multifocal retinopathy (cmr; gene: *BEST1*) [8], [9], rod-cone dysplasia type 1 (rcd1; *PDE6B*) and type 3 (rcd3; *PDE6A*) [23]–[26], progressive rod-cone degeneration (prcd; *PRCD*) [27], canine Leber congenital amaurosis (LCA; *RPE65*) [6], [7], cone-rod dystrophy (crd^SWHD^, *NPHP4*) [28], and achromatopsia/cone degeneration (ACHM/cd; *CNGB3*) [29], [30]. PRA-unaffected dogs were defined as Swedish Vallhund dogs over the age of six years who were examined by a board certified veterinary ophthalmologist and confirmed free of retinal disease.

Primers were designed to amplify two flanking microsatellites using Primer3 software (http://frodo.wi.mit.edu/primer3/) and the canine whole genome sequence assembly (CanFam2.0: http://www.ensembl.org/Canis_familiaris/; see **Table S2** for PCR amplification primers). An 18-bp extension sequence was added to the 5′-end of the forward primers to allow amplification of a fluorescently labeled third primer Fam-TGACCGGCAGCAAAATTG for visualization. Microsatellites were PCR-amplified using the pooling method: Two pools, one containing DNA from PRA-affected dogs, and one containing DNA from control dogs were made, using the exact same amount of DNA (**Table S3**). GeneScan LIZ-500 dye size standard (Applied Biosystems by Life Technologies, Carlsbad, CA, USA) was added to the PCR products, combined with Hi-Di formamide, and placed onto an ABI3730xl DNA analyzer (Applied Biosystems). The data was analyzed using Peak Scanner Software v1.0 (Applied Biosystems) and compared between the two groups. Possible PCR saturation was inspected to ensure that none of the amplifications had gone to completion. As the exact same quantity of DNA was used from each individual, the fragment sizes were able to be directly compared between pools. If differences were observed between case and control pools, individual samples were amplified separately using the same protocol. Minor differences were observed between case and control pools for *BEST1, CNGB3* and *NPHP4* genes, therefore the cases and controls were further amplified separately for these genes (**Table S3**). The individual microsatellite data was then analyzed for the presence of segregation under a recessive model.

### Evaluation of Serum Vitamin E Concentrations and Other Potential Risk Factors

Because some aspects of the retinal disease phenotype were remotely reminiscent of vitamin E deficiency [31], [32], serum vitamin E measurements were performed in a total of 19 dogs including normal and PRA-affected Swedish vallhund dogs as well as mixed breed colony dogs from the Retinal Disease Studies Facility at the University of Pennsylvania School of Veterinary Medicine. Following collection, serum samples were frozen until analysis at the Pennsylvania Animal Diagnostic Laboratory System Toxicology Laboratory at the University of Pennsylvania School of Veterinary Medicine. For sample preparation, vitamin E was extracted from the serum, filtered, and analyzed with a liquid chromatography system equipped with fluorescence detector (Shimadzu, Columbia, MD, USA). For quality control, serum with certified vitamin E concentration from the National Institute of Standards and Technology (NIST, Gaithersburg, MD, USA) was obtained and analyzed together with each batch of our canine serum samples. All vitamin E measurements were reported as the concentration of the most biologically active homologue, *alpha* tocopherol. A Wilcoxon rank sum test was performed to compare the values between groups.

In an attempt to identify any environmental risk factors contributing to the development and progression of PRA in Swedish vallhund dogs, a short questionnaire was distributed to dog owners and breeders. Topics of inquiry included husbandry practices, as well as the previous occurence of any systemic or ocular disease.

## Results

### Clinical Phenotype

**Figure 2:**
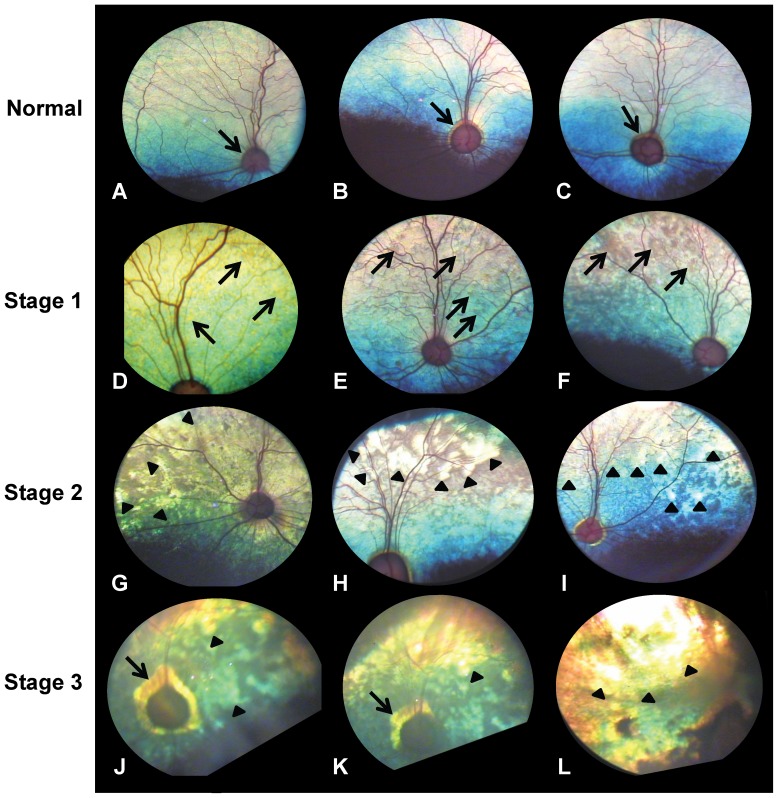
Clinical appearance of the ocular fundi of normal and affected Swedish vallhund dogs. (A–C) Normal appearance of the ocular fundus with the colored tapetum lucidum (responsible for eye shine), the well developed retinal vasculature, and the optic nerve head (arrows). It is not unusual to see a thin halo of altered reflectivity or *conus papillaris* around the optic nerve head as seen in B and C. (D–F) *Stage 1* is characterized by multifocal red or brown discoloration of the tapetal fundus (arrows). (G–I) In *Stage 2*, the retina showed signs of degeneration, indicated by multifocal, geographic thinning (arrow heads), beginning in the periphery and spreading throughout the tapetal fundus. (J–L) *Stage 3* is defined by a more diffuse retinal thinning recognized by the large, bright (hyperreflective) regions affecting most of the tapetal fundus (arrow heads). The *conus papillaris* also appears wider in *Stage 3* (arrows).

Between June 2005 and November 2011 we examined a total of 199 Swedish vallhund dogs. In addition, we evaluated eye examination data from certified veterinary ophthalmologists and eye panelists provided to us by the owners of an additional 125 Swedish vallhund dogs. In total, 113 of the 324 dogs examined exhibited a clinical phenotype consistent with Swedish vallhund retinopathy, resulting in an estimated disease prevalence of 34.9%. Based on all the available information, we identified a new retinal disease phenotype which appears to be breed specific since we are not aware of the presence of a similar disease phenotype in other dog breeds. By following individual affected dogs over time (**Tables 1** **and** **2**), we were able to define three distinct stages of the disease based on the severity of the clinical signs. The following phenotypic descriptions and numbers of affected dogs are based on the clinical examinations performed by the authors (AMK and PV) exclusively. *Stage 1*, the earliest stage with the mildest clinical signs, was characterized by a diffuse multifocal red or brown discoloration of the tapetal fundus (**Fig. 2D**–**F**). These abnormalities were seen in 34 dogs with a mean age (± standard deviation) of 4.3±3.7 years, but were noted as early as 1.9 months and as late as 17.8 years of age. There were no visible lesions in the nontapetal fundus. Dogs in *Stage 1* exhibited no clinical signs of vision loss. In *Stage 2*, the retina showed signs of degeneration, indicated by multifocal, geographic thinning, beginning in the periphery and spreading throughout the tapetal fundus (**Fig. 2G**–**I**). A total of 25 dogs were examined and found to have *Stage 2* disease. The mean age at diagnosis was 6.2±3.1 years but changes were seen as early as 1.1 and as late as 12.6 years of age. While the majority of dogs with these degenerative changes did not appear to have vision problems initially, some owners reported that their dogs exhibited mild to moderate signs of night-blindness as the areas of retinal thinning expanded. Seven dogs with a mean age of 12.2±2.6 years were diagnosed with *Stage 3* disease, characterized by more diffuse retinal thinning affecting most of the tapetal fundus (**Fig. 2J**–**L**). *Stage 3*-changes were observed as early as 9.2 years and as late as 15.4 years. These dogs suffered from loss of night-vision and severely impaired day-vision; some dogs were assessed as completely blind by their owners.

**Table 1:** Swedish vallhund dogs that showed progression with repeated examination.

Vallhund ID	Age at Stage 1 diagnosis (years)	Age at Stage 2 diagnosis (years)	Age at Stage 3 diagnosis (years)
**CSC**	2.2	3.4	_
**GRP**	3.9	4.9, 6.9, 9.1	_
**LHU**	0.6	2.0	_
**PCC**	1.5	3.7	_
**GBB**	3.9	4.9, 6.9, 9.1	_
**FEE**	_	9.6	13.4
**FJJ**	_	8.6, 8.7, 9.2	15.3
**FY**	_	4.6	8.3
**JMM**	_	11.4, 12.0	14.5
**FL**	_	5.3	12.6

At the authors' discretion, selected examinations performed by other certified veterinary ophthalmologists were included in the data analysis in order to provide a more complete picture of disease progression.

**Table 2:** Swedish vallhund dogs that showed no progression to subsequent disease stages with repeated examination.

Vallhund ID	Age at Stage 1 diagnosis (years)	Age at Stage 2 diagnosis (years)	Age at Stage 3 diagnosis (years)
**FHL**	0.8, 1.8	_	_
**JLG**	16.9, 17.4, 17.8	_	_
**JM**	10.4, 11.4	_	_
**KCY**	5.2, 7.4	_	_
**WAG**	1.9, 2.9	_	_
**CTG**	_	8.7, 11.5, 12.6	_
**HMAY**	_	4.9, 7.1	_
**KKB**	_	1.1, 4.1	_
**VM**	_	2.2, 4.0	_

In order to better understand the rate of disease progression, whenever possible, repeated examinations of specific dogs were performed over the 6.5-year period of the study. Although the precise duration that an animal spent in a particular disease stage could not be determined based on the voluntarily nature of the examinations and the widespread distribution of the study population across several continents, repeated examinations were used to provide a rough estimate of disease progression. When looking at dogs with multiple examinations, a total of five dogs were followed at *Stage 1* for a minimum period of 0.9 years and a maximum period of 2.3 years (mean of 1.2 years) without any signs of progression to subsequent disease stages (**Table 2**). One example of note is a dog that was repeatedly examined until the age of 17.8 years but never progressed beyond *Stage 1*. A total of four dogs were followed at *Stage 2* for a minimum period of 1.8 years and a maximum period of 3.9 years (mean of 2.7 years) without any signs of progression to subsequent disease stages. The discrepancies noted with respect to rate of disease progression among Swedish vallhund dogs suggest that genetic or environmental disease modifiers are likely to play a role in the pathogenesis. During the study period, a total of ten dogs showed progression to subsequent disease stages with repeated examination (**Table 1**). These dogs were followed for a minimum period of 1.1 years and a maximum period of 7.3 years (including other ophthalmologists' findings) with a mean of 4.0 years.

**Figure 3:**
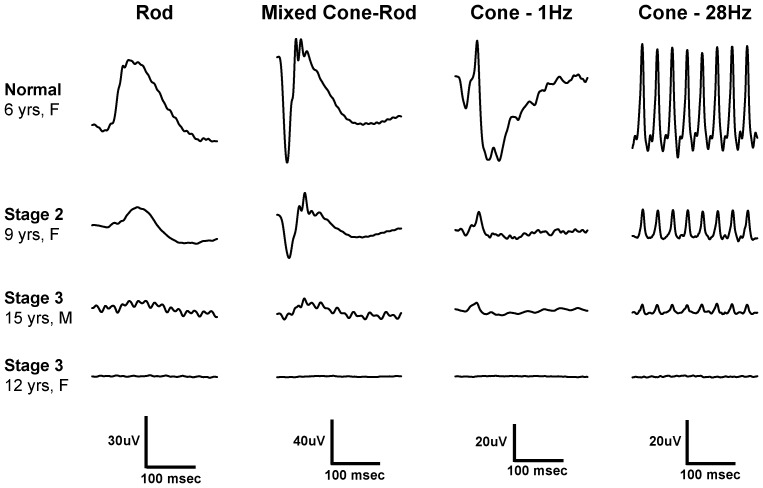
Standard electroretinograms (ERGs) recorded from normal and affected Swedish vallhund dogs. Compared to the ERG recordings of a normal dog (top row) both rod- and cone-mediated retinal functions are clearly reduced at *Stage 2*. ERG amplitudes are further decreased at *Stage 3* (third row), especially rod-mediated responses, and may not even be recordable in some affected dogs (flat lines in fourth row).

Evaluation of retinal function in 15 Swedish vallhund dogs (nine dogs at *Stage 2*, two dogs at *Stage 3*, and four normal control dogs) by electroretinography revealed a decrease of both rod and cone photoreceptor-mediated function in *Stages 2* and *3* (**Fig. 3**). While the small sample size did not permit a detailed statistical analysis, our recordings indicate that relative cone function may be lost at a slower rate than rod function; this would be consistent with the dog owners' observations that night-blindness precedes loss of day-vision at *Stage 3*.

Unrelated to the herein described phenotype specific to Swedish vallhund retinopathy, we also observed focal and multifocal inactive chorioretinal scars in a number of dogs (n = 14). These lesions were characterized by focal areas of pigmentation that were often surrounded by a hyperreflective halo due to retinal thinning (**Fig. S1**). These ‘bull's eye’ lesions are most consistent with multifocal acquired chorioretinopathy (MAC), a disorder previously reported in herding and working dogs [33]–[36]. According to information provided by the dog owners, at least some of the Swedish vallhund dogs with MAC-like lesions suffered from severe gastrointestinal parasitism early in life which may have resulted in multifocal chorioretinitis. In three Swedish vallhund dogs we found these MAC lesions combined with the breed-specific retinopathy. Two Swedish vallhund dogs that were not examined by the authors were diagnosed with retinal folds (n = 2, 0.85% of all examinations), a mild form of retinal dysplasia.

### Histologic Phenotype

**Figure 4:**
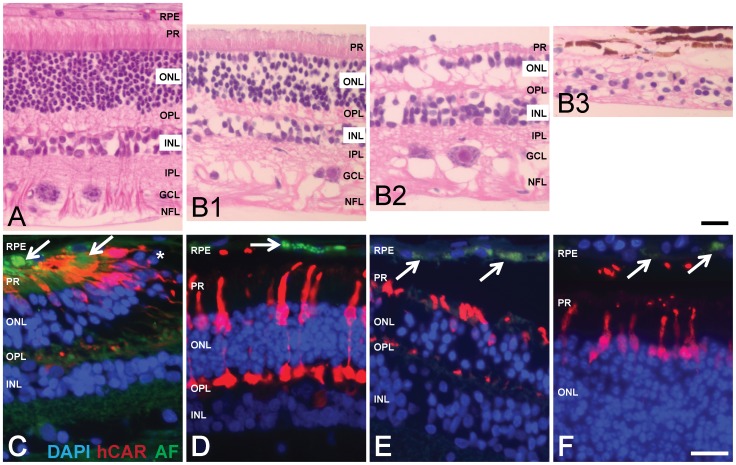
Histologic changes associated with Swedish vallhund retinopathy. (A) High quality section through a wildtype canine retina shows the normal retinal layers and architecture. (B) Different regions of the same 15-year old Swedish vallhund retina with *Stage 3* disease. Small islands of relatively normal appearing retina (B1) are surrounded by severely degenerated retina characterized by the loss of photoreceptors and ONL thinning (B2). Retinal architecture is completely lost with end-stage disease and severe retinal degeneration (B3). (C) At the transition between relatively well preserved and completely absent (*) retinal architecture, loss of photoreceptors (cones are labeled red with hCAR antibody) is associated with accumulation of autofluorescent material (green) in the subretinal space and within the RPE (arrows). (D) Accumulation of autofluorescent material (green) within the RPE can also be seen in a well preserved region of a 13-year old *Stage-2* affected retina (arrow), possibly indicating that increased autofluorescence may precede loss of photoreceptors. Accumulation of autofluorescent material within the RPE (arrows) can also be seen with other forms of PRA (E, prcd) or with normal aging (F, 11-year old normal canine retina) but to a much lesser extent than in affected Swedish vallhund dogs. Staining: H&E (A and B) and fluorescent immunolabeling (C–F). Cell nuclei are shown in blue with DAPI (C–F); hCAR, human cone arrestin; AF, autofluorescence. Calibration bars: 20 µm. Layers of the retina: RPE, retinal pigment epithelium; PR, photoreceptors; ONL, outer nuclear layer; OPL, outer plexiform layer; INL, inner nuclear layer; IPL, inner plexiform layer; GCL, ganglion cell layer; NFL, nerve fiber layer.

Because our study involved only privately owned dogs and no purpose-bred research animals, the availability of tissue samples for histologic examination was limited to eight eyes generously donated by owners. As such, these samples were typically obtained from older dogs that died of causes unrelated to the retinopathy. The retinal disease status was not always known at the time of death and tissue collection. Despite these limitations, the available tissues still provided valuable information about changes in retinal architecture in advanced disease which may contribute to a better understanding of the etiopathogenesis. Most valuable was the tissue sample from a 15-year old *Stage-3* retina with distinct regions of varying disease severity (**Fig. 4B**): Small islands of relatively normal appearing retina (**Fig. 4B** **1**) were surrounded by large areas of advanced retinal degeneration with loss of both rod and cone photoreceptors (**Fig. 4B** **2**). Regions with ‘end-stage’ disease showed severe retinal thinning with complete loss of normal architecture (**Fig. 4B** **3**). The transition between mildly and severely degenerated retinal regions was at times abrupt (**Fig. 4C**). The loss of photoreceptors was associated with the accumulation of autofluorescent material in the subretinal space and within the RPE (**Fig. 4C**–**D**). The presence of this material was more excessive than would be seen with other forms of PRA (e.g., prcd shown in **Fig. 4E**) or normal aging (**Fig. 4F**). We posit that this acid-fast positive, ceroid/lipofuscin-like material was likely the source of the red or brown discoloration clinically seen in the tapetal fundus and defining *Stage 1* of the disease. However, based on the available data, the presence of autofluorescent material does not provide us with specific clues about the cellular and molecular disease mechanisms resulting in photoreceptor loss and retinal degeneration. We suspect that the disease-related increase in autofluorescence may be able to be visualized in the living animal when proper imaging tools are used. As such, this will be one of our future goals.

### Pedigree and Candidate Gene Analysis

**Figure 5:**
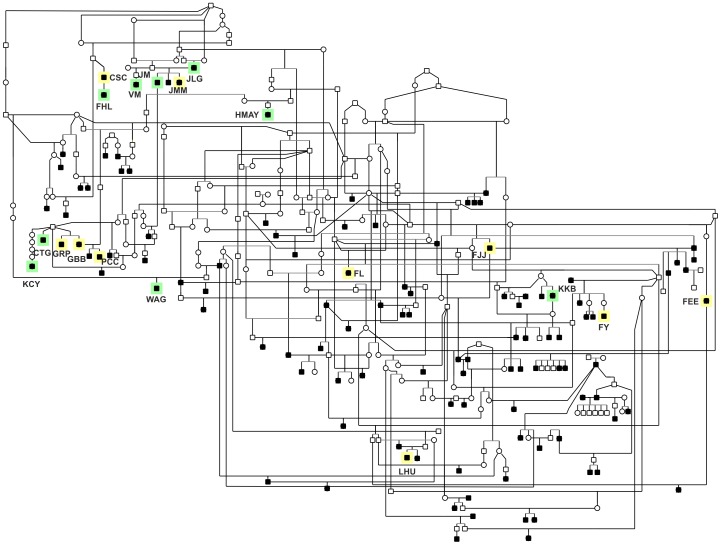
Large Swedish vallhund pedigree. This pedigree is composed of nearly 300 dogs, including 125 animals affected with the breed-specific retinopathy. Affected dogs listed in Tables 1 and 2 are highlighted in yellow and green, respectively. Squares, males; circles, females; white symbols, unaffected; black symbols, retinopathy affected.

Pedigree analysis of nearly 300 Swedish vallhund dogs, including 125 affected animals, indicates a clear genetic contribution (**Fig. 5**). While variability in the age of disease onset and incomplete pedigree information preclude us from drawing any definitive conclusions about the mode of inheritance, the available data is most consistent with an autosomal recessive pattern. This notion is supported by the lack of a sex predisposition, and the observation that affected animals can originate from the breeding of non-affected dogs.

In order to perform candidate gene screening, we collected blood samples from normal and affected Swedish vallhund dogs. Seven known canine PRA genes were tested for possible association: *BEST1* [8], [9], *PDE6B* and *PDE6A* [23]–[26], *PRCD* [27], *RPE65* [6], [7], *NPHP4* [28], and *CNGB3* [29], [30]. Flanking microsatellite markers were selected for each locus and tested in case and control pools for possible allelic differences. No PCR completion was detected in any of the pools. The affected dogs did not share common alleles in any loci of six candidate genes, excluding them as causative for the disease (**Table S3**). As both *PRCD* markers were monomorphic and uninformative, no conclusions could be drawn about a possible involvement of this gene in the disease process.

### Evaluation of Serum Vitamin E Concentrations and Other Potential Risk Factors

Because the fundic appearance in early stages of the Swedish vallhund retinopathy is reminiscent of vitamin E deficiency retinopathy [31], [32], [37], blood was collected and vitamin E (*alpha* tocopherol) levels were determined in three retinopathy-affected and six normal control Swedish vallhund dogs and compared to ten normal mixed-bred dogs housed in a well-controlled research colony. Overall, the serum vitamin E concentrations were lower in the Swedish vallhund dogs (18.8–44.5 µg/mL, median: 28 µg/mL) compared to the normal mixed-bred colony dogs (36.2–48.8 µg/mL, median: 40.7 µg/mL; p = 0.01, Wilcoxon rank sum test), but well within the previously described normal range for this species [31], [32], [37]. Except for one individual, the measurements of the affected animals (18.8, 22.9, and 26.2 µg/mL) fell within the range of the normal Swedish vallhund dogs (19–44.5 µg/mL; median: 29 µg/mL), making vitamin E deficiency an unlikely etiology for Swedish vallhund retinopathy.

## Discussion

Based on its inheritance patterns and the progressive nature of vision loss, the novel retinopathy observed in Swedish vallhund dogs appears to be a form of PRA. The phenotype of this disease is rather different from most known forms of PRA, with a multifocal rather than diffuse distribution of retinal degeneration. Furthermore, age of disease onset and rate of disease progression can vary considerably, even in closely related animals. Based on our observations gathered during ophthalmic examination of affected dogs, we propose three clinical stages of disease ranging from diffuse multifocal red/brown discoloration of the tapetal fundus without associated visual deficits (*Stage 1*), to geographic retinal thinning/degeneration with mild to moderate signs of night-blindness (*Stage 2*), to more diffuse retinal thinning/degeneration affecting most of the tapetal fundus and associated with night-vision loss and severely impaired day-vision (*Stage 3*). Paired with these clinical observations, functional and histologic examination of diseased retinas confirmed that this is a progressive retinopathy affects both rod and cone photoreceptors as well as the RPE. One of the most unique features of this disease is the excessive accumulation of autofluorescent material within the RPE, which may be the source of the initial discoloration noted within the tapetal fundus.

Our work to characterize this new canine retinopathy draws from well established collaborations between clinicians, geneticists, and dog breeders. Such an international effort was crucial considering that Swedish vallhund dogs are much fewer in numbers compared to other, more popular breeds. Our pedigree analysis included Swedish vallhund dogs examined in seven different countries, spanning three continents, and was highly suggestive of a genetic etiology and an autosomal recessive mode of inheritance. Because the affected dogs did not share common alleles, we excluded single, fully penetrant mutations in six known canine retinal disease genes: *BEST1* [8], [9], *PDE6B* and *PDE6A* [23]–[26], *RPE65* [6], [7], *NPHP4* [28], and *CNGB3* [29], [30]. However, dominant or incompletely penetrant mutations in any of these genes cannot be formally ruled out. Overall, the candiate gene analysis suggests that the Swedish vallhund retinopathy is genetically distinct from other forms of PRA, and our ongoing genome-wide studies have identified a new, unreported PRA locus in this breed (Ahonen et al., unpublished data).

The most striking feature of Swedish vallhund PRA/retinopathy is the individual variability in disease progression, even among related dogs and those raised in similar environments. While some of the affected dogs progressed to subsequent stages of disease with repeated examination, others did not progress beyond early stages despite advanced age. This is reminiscent of canine cone-rod dystrophy 1 (cord1-PRA, suspected *RPGRIP1* mutation) in the miniature long-haired dachshund, where genotype-phenotype discordance has been demonstrated within a genetically isolated population due to the presence of genetic disease modifiers [38]. Variability in disease phenotype caused by a particular mutation, either within one breed (e.g., X-linked PRA1) [39] or between multiple breeds (e.g., prcd) [40] has also been described in other types of PRA. Genetic disease modifiers that influence the severity of the disease phenotype also exist for other canine genetic disorders, such as Collie eye anomaly [41]. We suspect that genetic modifiers may be responsible for the variation in disease phenotype of PRA-affected Swedish vallhund dogs; as such, this will be a major focus of our future investigations.

Environmental disease modifiers may also play a role in determining onset and progression of PRA in the Swedish vallhund dogs. A classic example is the effect of light exposure on the progression of retinal degeneration in English mastiff dogs with autosomal dominant PRA [17]. Our attempt to identify environmental factors which may affect disease progression by questioning dog owners proved inconclusive. The observation of red/brown fundus changes at *Stage 1* and the accumulation of autofluorescent lipofuscin-like material within the RPE was remotely reminiscent of vitamin E deficiency retinopathy [31], [32], [37]. However, normal serum vitamin E (*alpha*-tocopherol) concentrations in a few affected Swedish vallhund dogs failed to support such a connection.

We posit that the accumulation of autofluorescent, lipofuscin-like material corresponds to the red/brown discoloration clinically seen in the tapetal fundus, and may precede the loss of photoreceptors. Future investigations using specialized, high-resolution *in vivo* imaging tools, such as optical coherence tomography and scanning laser ophthalmoscopy, will permit a much more detailed investigation into the source of the autofluorescence. Because this material appears to be more abundant in affected Swedish vallhund dogs compared to other forms of PRA with primary photoreceptor death or compared to the normal aging canine retinas, we suspect that the autofluorescent material is intimately associated with the disease-causing gene mutation. However, based on our available data, we are unable to conclude whether the Swedish vallhund disease primarily affects the RPE or the photoreceptors; both options are possible [42]. Pathologic accumulation of lipofuscin in the RPE is also a feature of several human diseases, including fundus flavimaculatus (Stargardt's disease) [43], but at this point it remains unclear which human condition is most closely modeled by Swedish vallhund PRA.

In summary, we describe a unique form of PRA in the Swedish vallhund dog. The novelty of this disease is not only based on the retinal disease phenotype, but also the variation in age of onset and rate of disease progression. An autosomal-recessive mode of inheritance appears most likely and mutations in several known retinal disease genes have been excluded. Genetic and/or environmental disease modifiers likely contribute to the disease phenotype, but hypovitaminosis E was excluded as a contributing factor.

## Supporting Information

Figure S1 **Multifocal acquired chorioretinopathy (MAC) in a Swedish vallhund dog.** The multifocal black spots in the tapetal fundus represent post-inflammatory scars. With a classic ‘bull's eye’ lesion, the larger black pigmented spot is surrounded by a bright, yellow zone or halo, a sign of severe, focal retinal thinning/degeneration.
(TIF)

Table S1 Primary antibodies used for immunohistochemistry.
(DOCX)

Table S2 Primers used to amplify microsatellite markers.
(XLSX)

Table S3 Association analysis of six known canine PRA genes in Swedish vallhund retinopathy. Eleven cases and eleven controls were pooled and genotyped for one to four microsatellite markers in each locus. The fragment sizes (bp) are marked in the table. The fact that the affected dogs do not share common alleles in any loci and same alleles were present in both cases and controls excludes these known PRA genes as candidates. For the *CNGB3_29_35.82* and *BEST1_18_57.49* markers, the pooled samples were individually genotyped to confirm the results. The *NPHP4* markers were genotyped separately in a different set of dogs.
(XLSX)
