# Correction: A Novel Form of Progressive Retinal Atrophy in Swedish Vallhund Dogs

**DOI:** 10.1371/journal.pone.0118128

**Published:** 2015-02-10

**Authors:** The PLOS ONE Staff

There is an error in the pedigree squares for the affected dogs in Figure 5. Please view the correct Figure 5 here.

**Figure 5:**
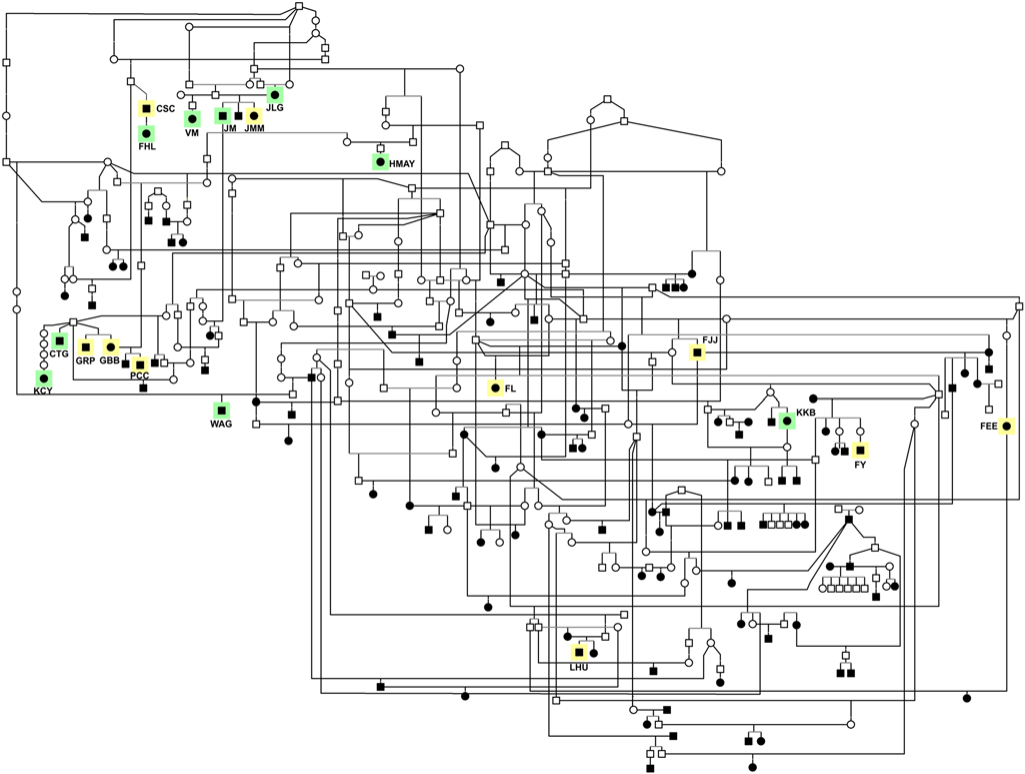
Large Swedish vallhund pedigree. This pedigree is composed of nearly 300 dogs, including 125 animals affected with the breed-specific retinopathy. Affected dogs listed in Tables 1 and 2 are highlighted in yellow and green, respectively. Squares, males; circles, females; white symbols, unaffected; black symbols, retinopathy affected.
