# Supplementary figures and images for: A Novel Form of Progressive Retinal Atrophy in Swedish Vallhund Dogs
Source: PLoS One. 2014 Sep 8;9(9):e106610. doi: 10.1371/journal.pone.0106610 (PMC4157785; doi:10.1371/journal.pone.0106610)

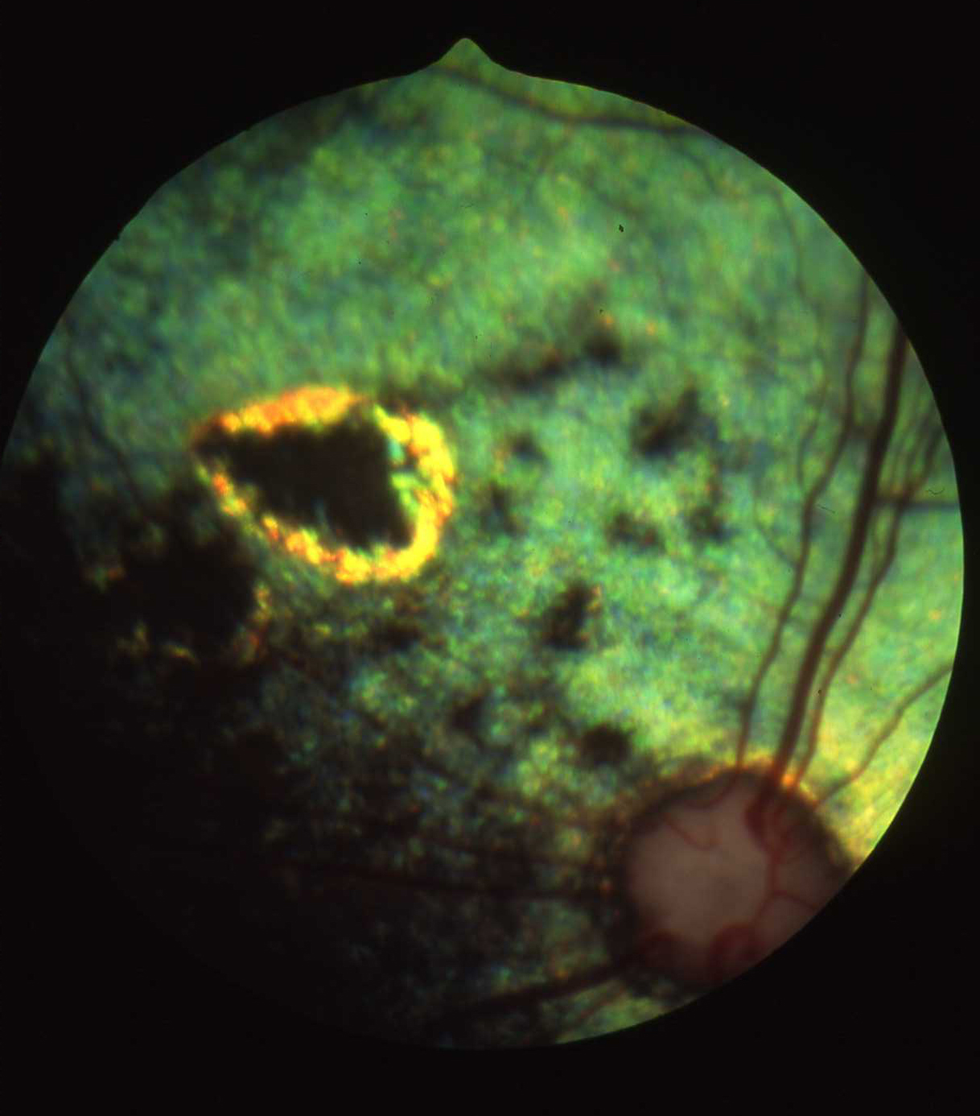

Supplement: Figure S1 — Multifocal acquired chorioretinopathy (MAC) in a Swedish vallhund dog. The multifocal black spots in the tapetal fundus represent post-inflammatory scars. With a classic ‘bull's eye’ lesion, the larger black pigmented spot is surrounded by a bright, yellow zone or halo, a sign of severe, focal retinal thinning/degeneration. (TIF) [file pone.0106610.s001.tif]
