# Supplementary material for: A Novel Form of Progressive Retinal Atrophy in Swedish Vallhund Dogs
Source: PLoS One. 2014 Sep 8;9(9):e106610. doi: 10.1371/journal.pone.0106610 (PMC4157785; doi:10.1371/journal.pone.0106610)
Supplement: Table S1 — Primary antibodies used for immunohistochemistry. (DOCX) [file pone.0106610.s002.docx]

**Table S1.** Primary antibodies used for immunohistochemistry.

| **Antibody** | **Host** | **Target Cell** | **Working Dilution** | **Source** |
| --- | --- | --- | --- | --- |
| HCar | Rabbit | Cone photoreceptors | 1:10,000 | Cheryl Craft |
| Rhodopsin | Mouse | Rod photoreceptors | 1:1,000 | Chemicon, Temecula, CA, USA |
| RPE65 | Mouse | Retinal pigment epithelium | 1:500 | Novus Biologicals |
| GFAP | Rabbit | Astrocytes, Müller cells | 1:1,000 | DakoCytomation, Carpinteria, CA, USA |
| Glutamine synthetase | Mouse | Müller cells | 1:20,000 | Chemicon, Temecula, CA USA |
| G_0_*alpha* | Mouse | ON (rod and cone) bipolar cells | 1:5,000 | DakoCytomation, Carpinteria, CA, USA |
